# Podoconiosis pathogenesis: renewed use of an historical archive

**DOI:** 10.1093/trstmh/try084

**Published:** 2018-09-18

**Authors:** Alexander Yardy, Anthony T Williams, Gail Davey

**Affiliations:** 1 Brighton & Sussex Medical School, University of Sussex, Falmer, Brighton, UK; 2 Brighton & Sussex Pathology, Brighton & Sussex University Hospitals, Eastern Road, Brighton, UK; 3 Department of Global Health & Infection, Brighton & Sussex Medical School, University of Sussex, Falmer, Brighton, UK

**Keywords:** Archive, histopathology, immunophenotype, lymph node, podoconiosis

In the light of the refreshed strategy of the Royal Society of Tropical Medicine and Hygiene (RSTMH), this commentary addresses a condition, podoconiosis, that sits firmly within the thematic areas prioritised by the RSTMH. Podoconiosis is unique, being both a neglected tropical disease (NTD) and a non-communicable disease. It is a condition that affects some of the most disenfranchised people and communities in the world and is only now beginning to receive the attention it deserves, in part through a programme of research that has explored disease distribution, burden, aetiology, sequelae, prevention and treatment. This brief commentary links two articles from the *Transactions of the Royal Society of Tropical Medicine and Hygiene* with Ernest Price’s recently rediscovered collection of archival materials and explains how these have shaped recent research into the pathogenesis of the NTD podoconiosis.

We have selected two articles written by Dr Ernest W. Price, who worked as a leprosy control officer in Ethiopia in the 1970s and 1980s. His curiosity was piqued by the large number of patients presenting to the leprosy hospital in Addis Ababa with leg swelling of undefined cause. Through the 1970s and 1980s he conducted a careful series of investigations of these patients and wrote more than 20 articles, including seven in the *Transactions.*^1–7^ He described the aetiology, pathogenesis, clinical progression and treatment of the disease, to which he eventually attached the term podoconiosis (from the Greek words for ‘foot’ and ‘dust’).

We have selected two articles, one from 1978^5^ and one from 1979.^6^ These compared the microparticles found in the lymph nodes of people with podoconiosis with those of unaffected barefoot comparators. The first study used light microscopy to identify birefringent particles within the lymph node macrophages and then electron microscope microanalysis to identify the most prominent elements in these particles.^5^ The second took the exploration a step further, through additional diffraction analysis. In both articles, groups of particles were reported within macrophages in the corticomedullary area, both within and outside lysosomes. Particles within the lysosomes were found in spherical clumps and were amorphous, whereas those outside were more often crystalline. Microanalysis confirmed the intralysosomal particles contained silicon (Si) and aluminium (Al), in the form of aluminosilicates or (in patients only) silica alone. The ratio of Si to Al was significantly different between patients and controls. Price suggested that the low pH of the lysosome might cause the crystals to disintegrate, becoming highly soluble aluminium hydroxide and insoluble silica. From this he suggested insoluble silica was the toxic agent in podoconiosis and that genetic differences in lysosome pH might cause differing susceptibility to developing the disease.^6^

These articles are of interest in themselves, but the event that caused us to go back to the *Transactions of the Royal Society of Tropical Medicine and Hygiene* archives was the ‘rediscovery’ in 2013 of several of Price’s notebooks, photographs, tissue blocks and slides. Ernest Price’s son, Michael, had stored his father’s effects since his death in 1990. When clearing his attic, he came across the boxes. He then discovered online that our group was doing research into podoconiosis, and so gifted the collection to Brighton & Sussex Medical School (BSMS). The BSMS Research Governance and Ethics Committee was alerted to the presence of the archive and offered guidance on its storage, particularly in relation to the Human Tissue Act. The Human Tissue Authority was also made aware of the archive, but agreed that further licensing was unnecessary given the HTA licence already held by BSMS.

Following cataloguing of the archive, a series of studies was planned, including investigation of the morphologic and immunophenotypic features of lymph node samples from podoconiosis patients using techniques unavailable to Price in the late 1970s. We summarise this investigation and then discuss the value of having access to the articles from the *Transactions of the Royal Society of Tropical Medicine and Hygiene* archive.

**Figure 1.**
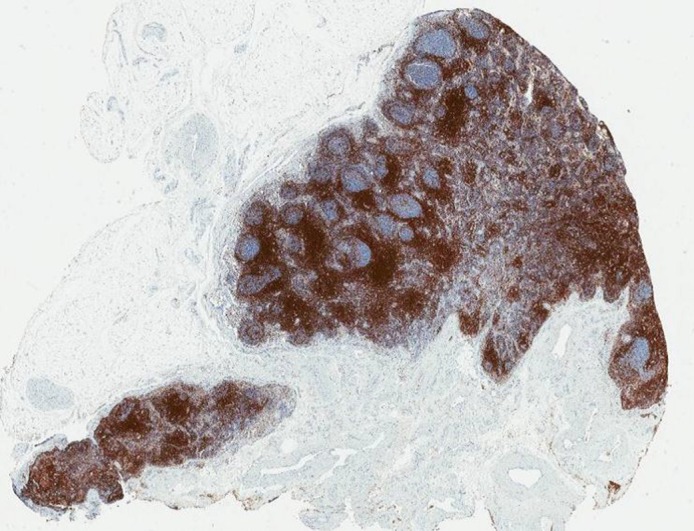
Lymph Node 607: Immunohistochemistry for CD3, marking the interfollicular T lymphocyte population and T lymphocytes surrounding the germinal centres.

We selected five lymph node blocks from the archive that were well preserved and clearly labelled as from a patient with podoconiosis. These were re-embedded, cut and stained with haematoxylin and eosin, periodic acid–Schiff (PAS), Perls’ Prussian blue, elastic van Gieson, Masson Fontana and a range of immunohistochemical stains. The extensive fibrosis described by Price^4^ was apparent in all nodes. All of the nodes we examined showed evidence of follicular hyperplasia, with an increased number of proliferative germinal centres. This is consistent with Price’s description of the nodes being enlarged^4^ and provides an explanation for the enlargement. An exaggerated lymphocyte population (Fig. 1) was seen in the paracortex sinuses of some of the nodes, though not to the same extent described by Price as ‘a sea of lymphocytes’.^5^ We were able to classify the immunophenotypic nature of these lymphocytes and found them to be predominantly CD4^+^ T lymphocytes. The characteristic particles Price described^5,6^ were found in all the nodes examined. These appeared as brown particulate matter, which we showed with special stains not to be melanin or iron. They were seen within macrophages in the cortex and medulla of the nodes, but unlike Price, we found none within macrophages with cytoplasm, which stained positively with PAS.

**Figure 2.**
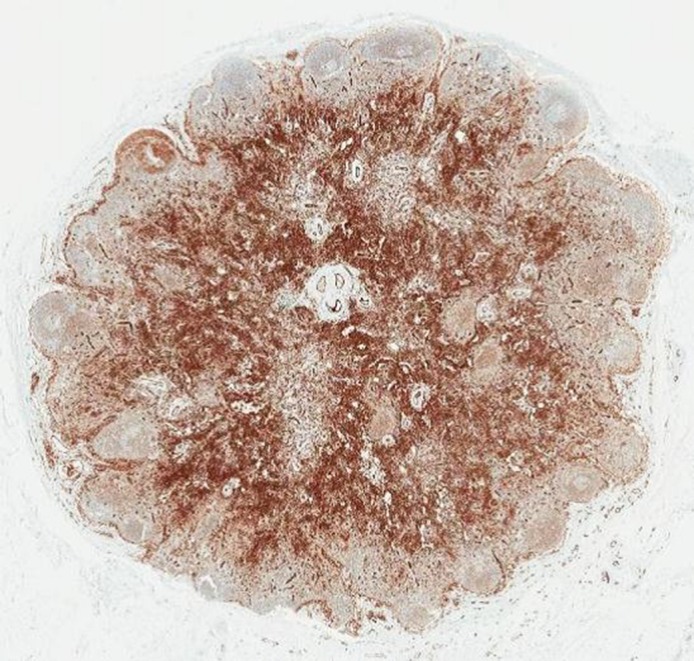
Lymph node 609: Immunohistochemistry for CD31, marking endothelial cells, highlighting the lymph node vasculature.

Serendipity enabled us to link articles published in the *Transactions of the Royal Society of Tropical Medicine and Hygiene* with a historical sample archive. Lymph node blocks and slides in the archive were labelled and their provenance clearly documented, enabling us to select appropriate samples. We found that most stains, including immunohistochemical stains, worked well on the archived material, although stains that included bleaching damaged the tissue, making interpretation difficult. We have been able to confirm most of Price’s descriptions and in some areas have taken them further, for example, by immunophenotyping the lymphocytic populations. We found potential discrepancies in relation to what Price identified as dilation of lymphatic vessels, which we identified as capillaries (Fig. 2), and acknowledge that tissue degradation may have affected the utility of PAS staining. We intend to build on Price’s work through the study of fine needle aspirates of lymph nodes of podoconiosis patients and to use more advanced techniques, such as energy dispersive spectroscopy, to determine the chemical composition of particles in archival and current-day samples.
